# What guidance is available for people with epilepsy wanting to participate in sport and exercise? A narrative review

**DOI:** 10.1016/j.jsampl.2026.100139

**Published:** 2026-05-07

**Authors:** Brittany Williams, Nathan Hill, Damien Cole, Sjaan Gomersall, Sean M. Tweedy, John Cairney, Jessica Hill

**Affiliations:** a School of Health and Rehabilitation Sciences, The University of Queensland, Brisbane, Australia; b School of Human Movement and Nutrition Sciences, The University of Queensland, Brisbane, Australia; c Health and Wellbeing Centre for Research Innovation, School of Human Movement and Nutrition Sciences, The University of Queensland, Brisbane, Australia; d The Queensland Centre for Olympic and Paralympic Studies, The University of Queensland, Brisbane, Australia

**Keywords:** Epilepsy, Sport, Exercise, Participation, Policy

## Abstract

**Background:**

This narrative review aimed to explore publicly available documents and policies that provide recommendations regarding safe sport and exercise participation for people with epilepsy. It also considered whether these documents addressed the needs of individuals with intellectual disability and other neurological conditions who have a co-occurring diagnosis of epilepsy.

**Method:**

A literature search was conducted across four electronic databases including PubMED, Embase, SPORTDiscus and Web of Science, accompanied by a hand search through Google Advanced Search. Documents were included if they were: 1) publicly available 2) produced by national and international government organisation, 3) published after 2016, 4) available in English, and 5) consisted of a full-text document.

**Results:**

A total of 11 papers were included. From the recommendations provided to the public four themes emerged, 1) general epilepsy advice, 2) guidance on what to do during participation, 3) considerations to avoid, and 4) activity specific recommendations. Eight of the papers provided recommendations to sport and exercise professionals, falling under three themes, 1) general epilepsy advice, 2) recommendations for sport and exercise professionals, and 3) key knowledge required to safely work with people with epilepsy. Of the 11 included papers, only four provided recommendations specific to people with disability.

**Conclusion:**

Findings revealed that whilst documents acknowledged risks, they did not provide strategies for risk mitigation or management to support participation in a variety of physical activity contexts. Additionally, the specific and often more complex needs of people with disability who have a co-occurring diagnosis of epilepsy were often overlooked.

**Key Points:**

**What is already known on this topic**.

• Sport and exercise are generally safe and beneficial for most people with epilepsy, yet participation remains low due to stigma, outdated risk averse messaging and lack of clear guidance.
• People with disability who have a co-occurring diagnosis of epilepsy experience additional challenges including more complex seizure presentations and increased safety concerns.

**What this study adds**.

• There is limited publicly available guidelines that provide practical recommendations to support inclusive participation in physical activity for people with epilepsy, particularly those with co-occurring disability.
• Current guidelines emphasise seizure risk over participation and neglect person centred strategies and collaborative planning for participation.

**How this study might affect research**.

• Findings highlight there is a need for publicly available co-designed, and contextually relevant resources that support safe participation in sport and exercise across a variety of fitness settings for people with epilepsy.

## 1 Introduction

Epilepsy is a chronic neurological condition that affects approximately 1%–50 million of the global population across all ages [[1], [2], [3], [4]]. Epilepsy is defined by the World Health Organization (WHO) as a condition characterised by recurrent seizures, where there may be brief lapses of attention or muscle jerks to prolonged convulsions and loss of consciousness [1]. Epilepsy includes a range of seizure types with differing presentations, frequency and severity, leading to varying levels of risk and impact on daily activities and participation [5].

Whilst epilepsy can affect anyone, individuals with intellectual disability and other neurological conditions (e.g. cerebral palsy or autism spectrum disorder) have a higher prevalence of co-occurring epilepsy and are also more likely to experience more severe and treatment-resistant forms of the condition, further increasing the complexity and potential safety risk for these individuals [6]. Individuals with intellectual disability and epilepsy may have cognitive, communication, and emotional regulation difficulties [7,8]. This may prevent reporting of potential seizure triggers, independently following safety precautions, or managing seizure triggers. For the purpose of this review, the term “individuals with disability” is used to refer to individuals with intellectual disability and other neurological or neurodevelopmental conditions.

Regular and sustained physical activity participation is widely recognised as a key contributor to positive health and wellbeing outcomes [9]. Research shows that regular and sustained physical activity participation for people with epilepsy (PWE) is linked to improved health and wellbeing outcomes such as improved physical and mental health, a reduction in associated comorbidities and improved overall quality of life [10]. While regular physical activity has not been conclusively shown to directly reduce seizure frequency, limited evidence suggests a potential association between sustained exercise participation and improved seizure outcomes in some individuals [11]. Physical activity has also been shown to support bone health, which is particularly important for PWE due to their increased risk of developing osteoporosis [4]. Despite these benefits, PWE continue to face multiple barriers to participation in physical activity and are less physically active when compared to the general population due to stigma, fear of seizure induction, overprotection and health professional's lack of knowledge and confidence to facilitate PWE within physical activity [12,13].

Sport and exercise are two important forms of leisure-time physical activity across the lifespan [14]. However, historically PWE were often discouraged from participating in sports and exercise due to concerns about safety, seizure risk and liability [15]. Given the importance of physical activity for health and quality of life and the concurrent evidence for low participation levels for people with disability and a co-occurring epilepsy diagnosis, understanding how to support participation has become increasingly important.

In 2016 the International League Against Epilepsy (ILAE) updated its guidance in a consensus report emphasising that physical activity is generally safe for most PWE [16]. Additionally, the World Health Organization's Intersectoral Global Action Plan (IGAP) on epilepsy and other neurological disorders outlined the importance of promoting full participation in life for PWE, including sport [17]. Despite these advancements, practical recommendations for supporting participation in physical activity remain inconsistent and do not sufficiently address the diverse needs of PWE across different sporting contexts. Although there is increasing evidence of the benefits of physical activity for PWE, many professionals and PWE lack clear guidance for inclusive and safe practice. Whilst it is acknowledged that people with disability and a co-occurring epilepsy diagnosis can experience additional complexities regarding managing their condition and safety [18], little is known about whether existing policies and guidelines address the unique needs of these individuals.

This narrative review aimed to explore what guidance is available for PWE wanting to participate in sports and exercise within publicly available documents and policies. This study also aimed to identify if and how the unique needs of people with disability and a co-occurring epilepsy diagnosis were addressed within these documents. To achieve these aims the following research questions were asked.

1) What are the current recommendations for epilepsy management within publicly available national and international sport and exercise guidelines?
2) What recommendations are provided specifically to people with disability who have a co-occurring epilepsy diagnosis?

## 2 Method

This paper involved the completion of a systematic search and narrative policy review for PWE and people with disability who have co-occurring diagnosis of epilepsy. This method was chosen to summarise and interpret the included documents, as the research required a qualitative review of diverse literature [19]. The review focused on publicly available documents, as these reflect the primary sources of information used by PWE, families and sport professionals to inform participation in sports and exercise.

### 2.1 Eligibility criteria

Documents were included if they met the following criteria: 1) consisted of publicly available policy documents, clinical practice guidelines, consensus statements, or position papers outlining recommendations for sport and exercise participation for PWE, 2) produced by national and international government organisation (e.g., Health Department), professional organisation (e.g., Sports Medicine Australia, Gymnastics Australia, etc.), international bodies (e.g., World Health Organisation), and non-government organisations and advocacy groups (e.g., Epilepsy Action Australia, etc.), 3) published after 2016 to align with the release of the ILAE consensus report, 4) available in English, and 5) consisted of a full-text document. Papers were excluded if they were 1) Peer reviewed research articles, editorials, commentaries, blogs, newsletters, or opinion pieces, published prior to 2016 to capture contemporary guidance following the 2016 ILAE position statement on sports and epilepsy, 2) not produced by a credible organisation as listed above, 3) not provided in English, 4) and/or when the full text was not available. The review was limited to English-language documents to reflect resources most readily accessible within English-speaking contexts.

### 2.2 Search strategy

A systematic literature search was conducted throughout May 2025 across four electronic databases including PubMED (n = 111), Embase (n = 325), SPORTDiscus (n = 31), and Web of Science (n = 290). Acknowledging that many policies and guidelines are published outside academic channels, a hand search was also conducted through Google (n = 17). An example of the search strategy including search terms applied for PubMed has been provided within Table 1. Documents arising from all searches were collated and imported into Covidence (2025), to allow for duplicates to be removed and to conduct screening and data extraction. Title and abstract screening were completed by authors BW and JH with authors meeting to discuss and resolve disagreements until consensus was reached. Authors BW and JH independently screened 100% of full-text articles with any article meeting the above inclusion criteria being included within the review.

**Table 1:** Example search strategy (PubMed).

(“Epilepsy"[Mesh] OR “epilepsy"[tiab] OR “epilepsies"[tiab] OR “epileptic"[tiab] OR “seizure disorder"[tiab] OR “seizure disorders"[tiab] OR “convulsive disorder"[tiab] OR “convulsive disorders"[tiab])
**AND**
(“Exercise"[Mesh] OR “Sports"[Mesh] OR “exercise"[tiab] OR “exercises"[tiab] OR “exercising"[tiab] OR “physical activity"[tiab] OR “physical activities"[tiab] OR “fitness"[tiab] OR “workout"[tiab] OR “work out"[tiab] OR “workouts"[tiab] OR “work outs"[tiab] OR “working out"[tiab] OR “sport"[tiab] OR “sports"[tiab] OR “sporting"[tiab] OR “recreational activity"[tiab] OR “recreational activities"[tiab])
**AND**
(“Guideline” [Publication type] OR “guidelines as Topic"[Mesh] OR “Consensus"[Mesh] OR “consensus development conference” [Publication type] OR “consensus development conferences as Topic"[Mesh] OR “rapid evidence assessment"[tiab] OR “rapid evidence assessments"[tiab] OR “guideline"[ti] OR “guidelines"[ti] OR “CPG"[tiab] OR “CPGs"[tiab] OR “consensus"[tiab] OR “recommendation"[tiab] OR “recommendations"[tiab] OR “best practice"[tiab] OR “position statement"[tiab] OR “position statements"[tiab] OR “protocol"[ti] OR “protocols"[ti])

### 2.3 Data extraction and analysis

Final papers were examined and date extracted by BW using an Excel spreadsheet (Microsoft, 2025). Data related to paper's country, sport/exercise activity, purpose/aim, and intended population was extracted. Additionally, a deductive thematic approach was used to analyse data related to recommendations provided to people with epilepsy against the two research questions [20]. To confirm the accuracy of the extracted information, BW and JH independently extracted data from 100% of the articles.

## 3 Results

After the removal of duplicates, 209 papers were screened for title and abstract, with 18 meeting the criteria for full-text review. At the completion of an independent full-text review, a total of 11 papers were included.

### 3.1 Paper characteristics

A full summary of the included papers has been provided within Table 2. Of the included papers, seven were published within Australia. Five were published by epilepsy advocacy organisations, five were published by either national or international sport and exercise organisations with one being published by a state government. In relation the target populations addressed within each paper, most (n = 7) were specific to PWE, with the remaining four addressing all people, however providing specific recommendations for PWE. Whilst the aims and purpose for each paper differed slightly, when addressing PWE, the purpose of all the included papers was to provide guidance to either PWE, or the people supporting PWE (i.e., coaches, family members, carers) regarding safe sport and exercise participation.

**Table 2:** Summary of included studies.

Publishing Organisation	Country	Title	Target Population	Activity	Purpose
International League Against Epilepsy (2016)	International	Categorization of physical activities and sports by risk in Epilepsy	All people with epilepsy	Sport	To classify sports into three levels of risk for people with Epilepsy ad bystanders.
Exercise and Sports Science Australia (2017)	Australia	Exercise right for kids - Condition factsheet: Epilepsy	Children with epilepsy	Exercise	To provide general recommendations regarding safe exercise participation for children with epilepsy.
International Life Saving Federation (2016)	Belgium	Medical position statement MPS - 17: Seizures and Epilepsy	All people with epilepsy	Aquatic activities	To provide general recommendations regarding safe participation in aquatic activities for all people with epilepsy.
Sports Medicine Australia (2017)	Australia	Safety guidelines for children and young people in sport and recreation.	Children and young people	Sport and recreation	To provide general recommendations regarding safe sport and recreation participation for children and young people.
Australasian College of Sport and Exercise Physicians (2017)	Australia/New Zealand	Australasian College of Sport and Exercise Physicians Curriculum and Tutorial Program (Version 6)	All people with epilepsy	Sport and exercise	To detail the scope, learning outcomes and competencies required for training and certifying sport and exercise medicine physicians in Australia and New Zealand.
Epilepsy Foundation (2019)	Australia	Physical activity and leisure	All people with epilepsy	Sport and exercise	To provide guidance to people with epilepsy, as well are caregivers regarding safe participation in sport and exercise.
Epilepsy Foundation (2019)	Australia	Living with epilepsy and cognitive disability: A guide for families, carers and support workers	People with epilepsy and co-occurring cognitive disability	Sport	To provide a general education resource for carers and family members of people with epilepsy and a cognitive disability.
New South Wales Department of Education (2020)	Australia	Requirements for all sport and physical activity	School aged children with epilepsy	Sport	To provide a list of requirements for the safe participation of sport and physical activity occurring within school events within NSW.
Exercise and sports science Australia (2021)	Australia	Pre-exercise screening system (PSS) for young people: User guide	Children and young people with epilepsy	Exercise	A pre-exercise screening tool to help identify young people with known disease, and/or signs or symptoms of disease, who may be at a higher risk of an adverse event due to exercise.
Epilepsy society (2022)	United Kingdom	Exercise and sport: Making choices about exercise and sport	All people with epilepsy	Sport and exercise	To provide general information relating to safe sport participation for people with epilepsy.
Epilepsy action (2025)	United Kingdom	Sport exercise and leisure activities	All people with epilepsy	Sport and exercise	To provide information on how people with epilepsy can participate in sport and exercise safely.

### 3.2 Research question 1: what are the current recommendations for epilepsy management within publicly available national and international sport and exercise guidelines?

A full outline of the specific recommendations provided to the general public, sport and exercise professionals (e.g. coaches, fitness instructors, exercise physiologists and safety personnel such as lifeguards) has been presented in Table 3.

**Table 3:** Recommendations for people with epilepsy wanting to participate in sport and exercise.

Date	Publishing organisation	Title	General recommendation	Recommendation to sport and/or exercise professionals
2016	International league against epilepsy	Categorization of physical activities and sports by risk in epilepsy	Group 1 - No significant additional risk to people with epilepsy or to bystanders: Athletics, bowling, most collective contact sports, curling, most team sports taking place on grass or court, nordic skiing, dancing, golf, racquet sports. Group 2 - moderate additional risk to people with epilepsy; no additional risk to bystanders: Alpine (downhill) skiing, archery, biathlon, triathlon, canoeing, modern pentathlon, collective contact sports involving potentially serious injury, cycling, fencing, gymnastics, horse riding, ice hockey, shooting, skateboarding, skating, snowboarding, swimming, waterskiing, weightlifting. Group 3 - high risk for people with epilepsy; for some sports, additional risk to bystanders: Aviation, climbing, diving, horse racing, motor sports, rodeo, parachuting, scuba diving, ski jumping, solitary sailing, surfing and windsurfing.	N/A
2017	Exercise and sports science Australia	Exercise right for kids - condition factsheet: Epilepsy	1) Before engaging in an exercise program consult your child's treating specialist, GP and accredited exercise physiologist, 2) Avoid your child's known seizure triggers, 3) Ensure appropriate medications are taken, 4) Ensure your child stays well hydrated and has a drink or snack to avoid hypoglycaemia, 5) If your child feels faint, lightheaded, nauseous or dehydrated, avoid exercise, 6) Ensure your child wears their medical alert bracelet, 7) If involved in water sports, ensure your child wears a life jacket.	N/A
2016	International life saving federation	Medical position statement MPS - 17: Seizures and epilepsy	1) Epilepsy carries increased relative risk of submersion and drowning. Children and adults should be advised of this increased relative risk, 2) Individuals with unstable, or potentially unstable epilepsy should avoid water activity until stability is re-established, 3) Epilepsy submersion and drowning risk is greatest in an identified high-risk group that includes those with frequent (more than one per year) seizures, those with unpredictable convulsive seizures, and/or those who have other disabilities. Extra precautions and attention are warranted. This patient group should avoid water activities, or should participate in clear, shallow, still water, with a (securely fastened) personal flotation device that will support an unconscious person, and they should be within arm's length of a capable support person. 4) Individuals with stable, controlled epilepsy and no other risk factors, who participate in recreational, instructional, and competitive water activities, should do so in supervised areas and with another capable person, 5) It may be helpful to provide absolute risks, so that persons with epilepsy and their advocates can make informed risk management decisions.	1) There are no higher levels of evidence studies on lifeguard patrol duties, deep water recreation, or competition participation by persons with epilepsy. Further research in this area may be helpful, 2) Lifeguard patrol duty and deep open-water recreation and competition participation for individuals with seizure disorders carries increased risk for the participant, co-workers, and the public. Sudden incapacity cannot be accepted, 3) There is no evidence on the seizure-free interval in swimming, lifesaving, or lifeguarding. Evidence is extrapolated from lower-level studies in other areas of similar concern (police, fire, military, driving licensing, and aviation). The seizure-free intervals below are extrapolated from other areas. They should be a guide; with medical reports, they can be used to determine safe participation. Further research in this area may be helpful, 4) Lifesaving training should be available to individuals with epilepsy who have confident seizure control, treatment programme compliance, knowledge of their risk, and a seizure-free period of at least 6 months. This interval may be longer if the participant has high-risk features. Proof of medical authorization, compliance, and stability may be requested. Consideration should be given to use of a safety craft to provide close supervision of the lifesaving learners with confident seizure control to assist with any unusual incidents that might arise, 5) If an organisation chooses to permit individuals with controlled epilepsy and no unexplained or unpredictable seizure activity to participate in lifeguard patrol duty or in deep-water recreation and competition activities, the organisation should require a seizure-free interval of more than one year prior to participation, 6) If an active patrol lifeguard, and/or a deep open-water recreation or competitive participant, has seizures with a clear medical cause, usual patrol and deep open-water recreation or competition activities should be suspended. If the lifesaving organisation chooses to permit resumption of activity, the organisation should require a seizure-free interval of more than one year prior to resumption, 7) Medical authorization should include as a minimum: a) A qualified medical doctor/Neurologist review, including consideration of the patient's level of knowledge of, and insight into, their medical condition; ability to self-manage the condition; compliance with physician-prescribed treatment, and willingness and ability to modify risk factors, b) A qualified medical doctor/Neurologist review of the lifeguard's job description, the range of responsibilities the lifeguard might assume to include: Extended water observation, swimming (distance and environment should be specified), oversight of large numbers of people who depend upon the lifeguard's vigilance, and any other responsibilities appropriate to the individual (local) assignment.
2017	Sports medicine Australia	Safety guidelines for children and young people in sport and recreation	Complete forms in detail	1) Coaches should be aware of any medication conditions of participants and how participation in sport might affect these adversely. 2) Epilepsy when medically supervised do not permanently preclude a child's involvement in sporting activity (but may at any given time exclude participation). 3) Coaches should ensure all participants complete a pre-season medical questionnaire and update this throughout the season as necessary. 4) Role of sports trainers is about risk management and risk management strategies extend to the acquisition and maintenance of emergency action plan. 5) Coaches should ensure key medical information about the participants is collected and taken into account before participation. 6) Coaches to be aware of the either (both hot and cold), young people are susceptible to extremes in temperature. Fluid replacement is important during sport. All children would be well h.
2017	Australasian college of sport and exercise Physicians	Australasian college of sport and exercise Physicians curriculum and tutorial program (version 6)	N/A	1) Having the knowledge to discuss the prevalence and key clinical features of the various types of epilepsy. 2) Understanding the possible triggers for an epileptic seizure. 3) Having the clinical knowledge to explain the pathophysiology of epilepsy. 4) Evaluate the preventative and management options from a sport's physician's perspective, including advice regarding what sport activities are contraindicated and which are safe to participate in. 5) Be able to identify the indications, contraindications, and special precautions regarding the persecution of physical activity for people with epilepsy. 6) Recognise the risk of diving with pre-existing condition of epilepsy. 7) Recognise the possible interactions between high-altitude exposure and epilepsy. 8) Be able to list specific precautions that should apply to children with epilepsy. 9) Perform a patient-centred assessment, including history and examination for a patient with epilepsy.
2019	Epilepsy foundation	Physical activity and leisure	1) Exercise has been found to not increase seizure activity in most people and can be an important part of epilepsy management. 2) Exercise contributes to good mental health which is particularly relevant to people living with epilepsy as they have a higher rate of depression and anxiety. 3) Exercise assists with maintaining a social life to counteract the feelings of isolation people with epilepsy experience (i.e. not being able to drive). 4) People with epilepsy can be component swimmers however seizures in water can lead to serious injury and death. 5) To stay safe int e water people with epilepsy should seek advice from their doctor about factors that could affect their safety in the water. Doctors may recommend not spending time in the water. 6) Only swim with someone that the person knows, who is familiar with the type of seizures and understands what to do if a seizure occurs and confident that they can provide first aid. 7) If at the pool or beach to let the lifeguard know. 8) Wear bathers or a swimming cap which is bright so that lifeguards can easily identify you, particularly if your seizures are uncontrolled. 9) People with uncontrolled seizures should wear a life jacket or buoyancy vest in the water. 10) Swim early in the day if seizures are triggered by heat or fatigue. 11) Do not swim or enter water if the person with epilepsy is feeling unwell, tired, or has missed medication or if they are experiencing any possible seizure signs. 12) Some sports and physical activities have higher risk for people with epilepsy and may put bystanders at risk. The level of risk is very individual and depends on several factors. Due to seizure types and frequency, it is recommended that certain activities only be undertaken in conjunction with someone under supervision. 13) Side effects of anti-seizure medication can impact ability and motivation to fully participate in physical activities. If this is the case the person should speak with their doctor. 14) If a person has undergone surgery for epilepsy they should avoid contact sports and activities where there is a chance they could hit your head. The exact time is negotiated with the treating doctor.	N/A
2019	Epilepsy foundation	Living with epilepsy and cognitive disability: A guide for families, carers and support workers	1) Common triggers for seizures include missed medication, feeling unwell or being overheated, dehydration and insufficient intake of water, low blood sugar, sleep deprivation, stress, bright, flashing lights. 2) A healthy lifestyle can assist with the medication management of epilepsy by undertaking appropriate exercise and stimulating activities. 3) All people have the right to be an active member of society. 4) There are several safety devices to assist with maintaining safety - medical ID bracelet, medical alert, assistive devices such as helmets, outward opening doors, bathing strategies, falls and tripping strategies, swimming strategies - having an observer or swimming companion trained in seizure first aid should always be present. 5) A safety assessment can be performed by an occupational therapist to assist with making recommendations and develop strategies to reduce risk and enhance safety. 6) It is recommended that family, carers and support workers receive training in understanding and managing epilepsy, seizure first aid, administration of emergency medication where prescribed.	1) There are tools to assist if you think a person is having a seizure and need to refer - these include observation statement or witness statement. 2) It is valuable to document seizure activity - recording how often seizures are taking place, how long symptoms last and how seizures are affecting behaviours. 3) It is important the person's epilepsy management plan is shared with staff. 4) If the person has emergency medication management plan, then staff need training to administer the emergency medication as required. 5) Understanding epilepsy-related risks and putting management strategies in place is a good way to assist the person to be supported and safe and is part of good management and governance practice. 6) Only people who have received person specific training for the administration of emergency medication for the individual should administer the medication.
2020	New South Wales department of education	Requirements for all sport and physical activity	N/A	1) When a student with epilepsy undertakes an activity a staff member should be present who has read the student's epilepsy management plan and is able to respond accordingly in the event of a seizure. 2) When a student with epilepsy participates in water sprots an individual observer (spotter) must be assigned to watch each student while in the water (meaning 1:1) supervision). This individual observer must be additional to the activity presenter, leader or instructor and can be a parent/grandparent etc.
2021	Exercise and sports science Australia	Pre-exercise screening system (PSS) for young people: User guide	N/A	1) To consider if exercise is a trigger for seizures. Consider what other triggers may come into effect with exercise such as overexertion, dehydration or low blood sugar levels. 2) Further screening is required if the young person has spent time in hospital for their medical condition within the last 12 months. Need to identify if the doctor or medical team has indicated any restrictions or potential impact of exercise. This information can be used to help determine if further screening is needed, if the client needs to be referred to an allied health or medical professional for additional assessments and as a basis for further questioning to help in the design of the client's exercise program. 3) If there is a current health or medical management plan the need to ensure it is current and the person has access to their medications at all times. 4) History of heat-related illness increases the risk of future events.
2022	Epilepsy society	Exercise and sport: Making choices about exercise and sport	1) If seizures are controlled the person may not feel the need to put safety measures in place. If seizures are uncontrolled there may be ways to make activity safer. 2) One way to think about safety is to do a risk assessment - this looks at what are the possible risks for anyone doing the activity and what the risks may be for the person with epilepsy and how to make them safer. 3) A person understanding their epilepsy can help decide what type of exercise or sport suits them - based off seizure triggers and telling other people how they can help if a seizure occurs. 4) Exercise can reduce the number of seizures a person may have. 5) A person may feel like not doing exercise because they are tired due to seizures or from side effects from medication however, or concern that they will hurt themselves during exercise if a seizure was to occur however research has shown regular session of aerobic exercise can result in a reduction of number of seizures. 6) To start exercising short, regular sessions of activity are recommended such as short walks. 7) Drink water, diluted fruit juice during exercise to replace fluids and body salts. 8) Do not exercise after a meal. 9) Walking with a friend means that they can help the person with epilepsy if a seizure occurs. If the person with epilepsy walks alone consider using well-known routines, taking a mobile phone and carry a medical ID. 10) Relaxing activities can be forms of exercise however extreme breathing techniques in yoga require additional care. 11) A person may want to speak to the GP or specialist about the risks of the risks, think about what may help make the sport safer (wearing a helmet, not walking near water or busy roads) or contact sport's governing body for information and advice on safety. 12) Water sports can be made safer by wearing a life jacket and having someone with the person who knows how to respond if you have a seizure. 13) Swimming in the pool where there is a lifeguard is generally safer than swimming in sea or open water. Swimming at quieter times makes it easier for someone to see the person with epilepsy. The person can tell the lifeguard at the pool how to help if they have a seizure. 14) Most sports, including contact sports like football, hockey, basketball and rugby have not been shown to increase chance of someone having a seizure however it is recommended the person tells their coach or someone on the team about their epilepsy and give them first aid information. 15) Extreme sports it is recommended the person speak to the doctor and sport's governing body. 16) Combat sports that involve blows to the head are not recommended for people with epilepsy. 17) It is recommended that when horse riding it is done with someone who knows what to do if you have a seizure. 18) Under the equality Act 2010 people with epilepsy are protected to have the right to use leisure facilities	1) Within water sports having a coach or instructor who knows how to responds if a seizure occurs
2025	Epilepsy action	Sport exercise and leisure activities	1) Being active and exercising can have health and wellbeing benefits. For some people when they are active, they are less likely to have seizures. 2) A small number of people find that doing strenuous exercise increases their risk of having seizures. 3) Equality laws in the UK prevent discrimination in sports and leisure activities however some sport activities have a governing body around safety and medical conditions. 4) You can lower risk by doing a safety check and putting safety measure in place. Some people use driving rules for epilepsy to decide how safe they feel doing a particular activity (i.e. if you have had a tonic clonic seizure you can't drive for a year). 5) Avoid exercising during times that you know may trigger a seizure or avoid exercise if you did not get a good night's sleep. 6) People with epilepsy may not need to share their condition if it is unlikely to affect their safety or others. 7) A person with epilepsy can share information such as what epilepsy is, what type of seizures they have, how to help if they have a seizure.	If the sporting club is required to do a risk assessment and make reasonable adjustments this should be done with the person not for them.

#### 3.2.1 Recommendations to the general public

From the recommendations provided to the general public four themes emerged, 1) general epilepsy advice, 2) guidance on what to do during exercise, 3) considerations to avoid, and 4) activity specific recommendations. Four of the 11 papers provided general epilepsy advice relevant to sport and/or exercise participation. Specifically, this advice included the benefits of sport and exercise for PWE, such as overall health and wellbeing benefits, as well as the potential for reduced seizure activity, potential risks of sport and exercise participation, common seizure triggers, as well as the rights of PWE in relation to sport participation [[21], [22], [23], [24]].

Seven of the included papers provided specific recommendations outlining safety strategies for participation of what PWE should do, and precautions to take if they were wanting to participate in sport and/or exercise. Most suggested gaining clearance from a health or medical specialist prior to participation [[21], [22], [23],^25^]. Five addressed specific actions or behaviours PWE should take to improve their safety such as staying hydrated and fuelled to reduce their seizure risk, as well as communicating their diagnosis to relevant sport and exercise professionals either by wearing a medical alert bracelet or by providing relevant documentation [[22], [23], [24], [25], [26]]. Whilst most recommendations related to sport and exercise activities more generally, four highlighted specific precautions to take if engaging in water sports such as wearing a life jacket, wearing bathers or a swimming cap with bright colours making it easy for lifeguards to identify them, and swimming in quieter times with at least one person present [[22], [23], [24], [25], [26]].

Five of the papers included within this review also provided recommendations on things to avoid such as avoiding seizure triggers, avoiding participation if the individual is feeling faint, light headed, nauseous or dehydrated, if the individual's epilepsy is unstable, or if the individual did not get a good night sleep [[23], [24], [25], [26], [27]]. These precautions were specifically highlighted for those participating in water sports or combat sports.

Finally, one paper identified specific sporting activities that were considered as low risk (e.g., athletics, bowling, etc), moderate risk (e.g., archery, cycling, etc), and high risk (e.g., climbing, surfing) for PWE. No further information was provided regarding how to mitigate these risks or whether PWE should or should not participate within these specific activities.

#### 3.2.2 Recommendations to sport and exercise professionals

Eight of the included papers provided specific recommendations to sport and exercise professionals with these recommendations falling under three themes. Theme one provided general epilepsy advice, whilst theme two provided recommendations for what sport and exercise professionals could do. Theme three highlighted key knowledge required by sport and exercise professionals to safely involved in training, supervising, or overseeing physical activity participation for PWE. Within theme one, one paper provided general epilepsy advice to sport and exercise professionals [27]. Specifically, this paper highlighted that whilst there was no literature suggesting that PWE should not participate in open water swimming or lifeguard patrol duties, further research was required.

Seven of the eight papers included specific recommendations for sport and exercise professionals supporting physical activity participation amongst PWE. Whilst all differed in how the information was worded and presented, all seven highlighted the importance of sport and exercise professionals having knowledge of the person's epilepsy, seeking medical clearance that they were safe to participate and completing appropriate risk assessments, and providing close supervision during participation within water sports [21,[23], [24], [25], [26], [27], [28]].

Two of the included papers highlighted specific knowledge sport and exercise professionals must have if they were to safely support physical activity participation for PWE [21,29]. This knowledge consisted of an understanding of the condition, potential risks and precautions, and training and administration of emergency procedures.

### 3.3 Research question 2: what recommendations are provided specifically to people with people with disability who have a co-occurring epilepsy diagnosis?

An outline of the specific recommendations provided to people with disability is presented in Table 4. Of the 11 included papers, only four provided additional recommendations specific to people with disability with all providing unique advice. For example, the International Life Saving Federation [27] identified people with disability with a co-occurring epilepsy diagnosis as a ‘high-risk’ group requiring additional precautions including increased restrictions and safety measures when participating in water activities. Sports Medicine Australia [26] recommended that sport and exercise professionals working with people with disability should ensure that appropriate activity adaptations are put in place prior to athlete participation. The Australian College of Sport and Exercise Physicians [29] provided advice specifically related to para-athlete travel to events, ensuring that all medical and clinical considerations had been made. Finally, the Epilepsy Foundation [22] discussed the increased risk to those with a cognitive disability participating in sport including increased seizure frequency and severity.

**Table 4:** Recommendations for people with disability with a co-occurring epilepsy diagnosis regarding safe sport and exercise participation.

Date	Publishing organisation	Title	Disability specific recommendation
2016	International league against epilepsy	Categorization of physical activities and sports by risk in epilepsy	N/A
2017	Exercise and sports science Australia	Exercise right for kids - condition factsheet: Epilepsy	N/A
2016	International life saving federation	Medical position statement MPS - 17: Seizures and epilepsy	1) Epilepsy submersion and drowning risk is greatest in an identified high-risk group that includes those who have other disabilities. Extra precautions and attention are warranted. This patient group should avoid water activities, or should participate in clear, shallow, still water, with a (securely fastened) personal flotation device that will support an unconscious person, and they should be within arm's length of a capable support person. 2) Individuals with chronic disabilities who are intellectually and neurologically able, and medically stable should be encouraged to undertake swimming and lifesaving training,
2017	Sports medicine Australia	Safety guidelines for children and young people in sport and recreation	1) Any limitation should be referred to a qualified sports first aider, sports trainer or medication professional for management. 2) Coaches should consider individual needs and adapt/modify activities if required.
2017	Australasian college of sport and exercise Physicians	Australasian college of sport and exercise Physicians curriculum and tutorial program (version 6)	Registrars to have experience in plan to travel overseas with a group of athletes (consider pre-departure planning, transit arrangements etc) and prepare a list of what medical supplies to take with them on the plane and in their medical bag. Prepare a list of important administrative and clinical considerations for a specific event with para-athletes.
2019	Epilepsy foundation	Physical activity and leisure	N/A
2019	Epilepsy foundation	Living with epilepsy and cognitive disability: A guide for families, carers and support workers	1) Understanding a dual diagnosis - people with cognitive disabilities are at a higher risk of developing epilepsy. People with epilepsy and a cognitive disability tend to have more severe, difficulty to control seizures and an increased risk of preventable death. 2) The impact of epilepsy on the person with a cognitive disability and their family is often far greater than the seizure itself. 3) It is important that people living with epilepsy and a cognitive disability have a much information to feel safe and supported to make informed choices. 4) Excitement over seeing a favourite support person can be a seizure trigger. 5) Encouraging people living with epilepsy to engage in social and leisure activity is helpful to enhance physical health, mental health and wellbeing. 6) The person should be supported to engage in activities that interest and appeal to them however some activities may be more hazardous (e.g. swimming) so choose activities that provide a reasonable degree of safety. 7) Not all community and sporting groups have experience with epilepsy and because of this the person, family and carers may need to meet with staff to develop individualised safety strategies. 8) People who live with epilepsy and a cognitive disability are at a greater risk of developing mental health issues and workers can help identify behaviours and symptoms which may indicate the person is experiencing mental health issues.
2020	New South Wales department of education	Requirements for all sport and physical activity	N/A
2021	Exercise and sports science Australia	Pre-exercise screening system (PSS) for young people: User guide	N/A
2022	Epilepsy society	Exercise and sport: Making choices about exercise and sport	N/A
2025	Epilepsy action	Sport exercise and leisure activities	N/A

## 4 Discussion

Research has consistently found that PWE are less physically active when compared to people without epilepsy [13]. Barriers identified in the literature include concerns that exercise may trigger seizures, medication side effects and a lack of clear advice from healthcare professionals, as well as broader psychosocial factors such as limited social support, low motivation and fatigue. [[30], [31], [32]]^,^ Additional barriers have been reported among individuals using neurostimulation devices, including uncertainty regarding device safety and malfunction [33]. It is important to note that exercise-induced seizures are reported in only 1–2% of PWE; however, it remains unclear whether this prevalence differs among people with disability with a co-occurring epilepsy diagnosis [34]. Findings from this review revealed that while there is a presence of guidelines available, the documents lacked actionable strategies for managing seizure risk and did not address the diverse context in which sports and exercise occur. There was also little consideration for the added complexities that people with disability and a co-occurring epilepsy diagnosis may experience.

In relation to research question one which explored what recommendations were provided to PWE to guide their safe sport and exercise participation, the majority of the current available guidelines adopted a deficit focused, risk adverse approach [[21], [22], [23], [24], [25], [26], [27]]. These recommendations had a focus on PWE gaining a medical clearance to establish whether or not they were “safe” to participate rather than, or in addition to, providing guidance on what safe participation might look like. This approach may inadvertently exacerbate current barriers to participation. Literature suggests that PWE refrain from participating in sports activities due to the fear of risk of injuries, fear of physical exercise inducing seizures, stigmatisation, and lack of efficient medical advice [35,36]. The focus on whether the individual is ‘safe’ without guidance on what safe participation might look like further contribute to fear and stigmatisation. None of the 11 papers contained the following recommendations put forth by the ILAE report that after 12 months of seizure freedom or resolved epilepsy, PWE may practice and compete in all sports [16].

Most papers suggested gaining clearance from a health or medical specialist prior to participation [[21], [22], [23],^26^]. However, literature highlights gaps in medical professional knowledge [37]. A recent study published in 2022 found that whilst 92.5% of neurologists surveyed across 16 different countries supported physical activity for PWE, only 40% were aware of the ILAE recommendations and 35% had no information about physical activities for PWE [24]. One paper included within this review [22] recommended an occupational therapist to assist with recommendations to reduce risk and enhance safety. Similar to medical professionals, literature highlights a gap in knowledge of allied health professionals when working with PWE [38]. For example, 42% of health professionals endorsed harmful outdated seizure practices, over one-third lacked vocational epilepsy training, and more than half underestimated seizure fatality risks, demonstrating the critical need for enhanced epilepsy education amongst allied health professionals [38].

Several of the available guidelines placed a particular focus on the risks associated with water sports and the need for additional precautions. [[21], [22], [23],^28^]. Notably most lacked guidance on risk mitigation strategies highlighting the critical gap in between risk identification and practical management solutions. Furthermore, there is a notable absence of guidance for land-based sports and exercise activities.

When discussing the specific risks to PWE participating in sport and exercise, all papers identified the potential for seizures. Capovilla et al. [16] suggested that when advising on sport participation, approaches to seizure management should be individualised whilst considering the following factors: probability of seizure occurring, the type and severity of seizures, seizure precipitating factors, the usual timing of seizure occurrence and the person's attitude towards accepting some level of risk. Despite these recommendations being available within the research, there was a lack recommendations for identifying individualised assessment and intervention strategies in the guideline documents identified in the current review.

In addition to risk of seizures, common anti-seizure medications used to treat epilepsy may also impact physical activity participation due to the potential side effects (e.g., fatigue, sleepiness, depressive mood, psychosis, anxiety or aggression and visual disturbances) [[39], [40], [41]]. Pattnaik et al. [42] reported that women with epilepsy undergoing polytherapy engaged in lower levels of physical activity compared to those receiving monotherapy. Within the included papers, there was minimal acknowledgement of these medications or reference to strategies to manage the common psychiatric and behavioural side effects associated. This highlights the need for interdisciplinary (neurologists, mental health professionals, and allied health providers) co-design with stakeholders and PWE to inform development of sports and exercise recommendations that recognise medication side effects.

In contrast to the inconsistencies and risk-averse framing seen in sport and physical activity recommendations, other areas of life for PWE are guided through a participation lens. In educational settings, the ‘Managing students’ health support needs at school’ procedure outlines the structured measures Australian State Schools must implement to ensure that students with health conditions, including epilepsy, can participate safely and fully in all aspects of school life [43]. These include the development of individualised health support plans to help school staff respond appropriately to seizures and provide necessary support to promote student safety and inclusion [43]. Although literature on epilepsy management and participation exist, particularly within medical and allied health contexts, many of these are either not publicly accessible or have not been meaningfully integrated into sport and physical activity policy documents. As a result, the practical application of epilepsy-specific knowledge in physical activity settings remains limited.

The documents identified in this review included minimal information on managing epilepsy in the context of a co-occurring disability. Whilst four of the total 11 papers provided disability-specific recommendations these were limited in scope and information to support participation. For example, within these papers people with disability and epilepsy were characterised as “high risk” [21,27]. However, no additional information was provided regarding strategies to support safe participation. The interplay of disability and epilepsy creates compounding challenges, as individuals with disabilities not only have higher epilepsy prevalence but also experience more severe and medication resistant forms of epilepsy [6]. Mahler et al., [18] found that PWE with co-occurring conditions were at a higher risk for injuries and accidents which further highlights the need for tailored recommendations rather than general risk identification. Whilst no research has been conducted exploring the physical activity rates of people with disability with a co-occurring epilepsy diagnosis, it is well established that both PWE and people with disability have significantly lower physical activity participation rates when compared to the general public [13,44]. Further research is required to establish sport and exercise recommendations that appropriately address the specific needs of both these populations, and must incorporate lived experience to ensure the development of inclusive and practical resources.

To our knowledge, this study was the first to provide a review of the current publicly available guidelines supporting the safe sport and exercise participation of PWE. It is acknowledged that this review was limited by several constraints. This review was limited to four electronic databases, English-language documents published after 2016, and focused on PDF-format documents during the hand search. One non-PDF document was included due to its high relevance and applicability to the review topic. As such, the search strategy may have missed relevant guidelines from non-English speaking countries or web-based recommendations presented in other digital formats. A further limitation is the exclusion of non-public or restricted-access documents, which may have led to the omission of relevant internal policies or guidelines used in practice but not freely accessible to the public.

### 4.1 Implications for future research and practice

Findings from this narrative review suggest a lack of practical policy documents supporting PWE and people with disability with a co-occurring epilepsy diagnosis to participate in sport and exercise. Many of the recommendations focused on medical clearance and risk identification without offering solutions for adapting activities or managing risk within specific sport or exercise contexts. The lack of clear guidance may result in overly cautious approaches that limit participation opportunities, or conversely, result in inadequate safety in some contexts. Future research should focus on the development of co-designed guidelines in sports participation that are applicable across diverse sporting environments and contexts. Future development of guidelines also needs to address the needs of people with disability with a co-occurring epilepsy diagnosis. Additionally, none of the identified documents explicitly considered sex or gender, which limits the inclusivity of the recommendations.

## 5 Conclusion

This narrative review aimed to explore publicly available guidelines and policy documents which provide guidance to PWE and/or sport and exercise professionals on safe sport and exercise participation. It also sought to identify if and how the needs of people with disability who have a co-occurring diagnosis of epilepsy were addressed within these documents. Findings revealed, there was a lack of guidance and practical strategies to support participation in a variety of physical activity contexts. Additionally, the specific and often more complex needs of people with disability who have a co-occurring epilepsy diagnosis were largely overlooked. To better support participation for PWE in physical activity there is a need for clearer publicly available guidelines that are tailored and appropriate across a range of sporting environments.

## Funding

This work was supported by the University of Queensland New Staff Grant. The funding body had no role in the study or publication process.

## Declaration of competing interest

The authors declare that they have no known competing financial interests or personal relationships that could have appeared to influence the work reported in this paper.
